# Combined clinical and MRI-based radiomics model for predicting acute hematologic toxicity in gynecologic cancer radiotherapy

**DOI:** 10.3389/fonc.2025.1644053

**Published:** 2025-08-08

**Authors:** Lumeng Luo, Jiahao Wang, Hongling Xie, Bingxin Chen, Hui Wang, Qiu Tang

**Affiliations:** ^1^ Department of Radiation Oncology, Women’s Hospital, Zhejiang University School of Medicine, Hangzhou, Zhejiang, China; ^2^ Zhejiang Provincial Key Laboratory of Precision Diagnosis and Therapy for Major Gynecological Diseases, Women’s Hospital, Zhejiang University School of Medicine, Hangzhou, China; ^3^ Department of Gynecologic Oncology, Women’s Hospital, Zhejiang University School of Medicine, Hangzhou, Zhejiang, China

**Keywords:** hematologic toxicity, radiomics, gynecologic cancer, magnetic resonance imaging, radiotherapy, machine learning

## Abstract

Acute hematologic toxicity (HT) remains a critical dose-limiting complication in gynecologic cancer patients undergoing pelvic radiotherapy, particularly when combined with chemotherapy. Early prediction of severe HT could inform personalized management and minimize toxicity. We developed and validated a predictive model integrating clinical parameters and radiomic features, evaluating five machine learning approaches. Clinical data, dosimetric parameters, and pelvic bone marrow radiomic features extracted from MRI and CT images were analyzed. Feature selection was performed using LASSO and random forest algorithms, followed by comparison across multiple classification models. In the independent test set, the combined clinical and MRI-radiomics model showed superior predictive performance (AUC=0.927, accuracy=85.5%, sensitivity=92.3%, specificity=66.7%) compared to clinical-only (AUC=0.703), MRI-only (AUC=0.925, but low specificity of 38.1%), and CT-only models (AUC=0.54). The model performed notably better in patients receiving concurrent chemoradiotherapy. Key predictors included baseline hemoglobin, white blood cell count, bone marrow dosimetry, and MRI-derived texture and fat fraction features. Integrating clinical data with MRI-based radiomics provides a robust approach for predicting acute HT, potentially guiding personalized management strategies and improving safety during gynecologic cancer radiotherapy.

## 1 Introduction

Acute hematologic toxicity (HT) remains a significant limitation in pelvic radiotherapy (RT), particularly in patients undergoing concurrent chemoradiotherapy (CRT) for gynecologic and other pelvic malignancies. Severe (grade ≥3) HT occurs in approximately 20–25% of patients receiving pelvic CRT, often causing treatment delays or chemotherapy dose reductions, potentially compromising oncologic outcomes (1, 2). Hematopoietic marrow within pelvic bones represents a substantial fraction of the body’s marrow reserve, making its sparing essential for maintaining blood cell counts during treatment (3, 4). Thus, accurate pre-treatment prediction of severe HT is critical to enable tailored interventions and avoid treatment interruptions.

Currently, clinical and dosimetric factors serve as the primary basis for predicting HT risk, including concurrent chemotherapy, patient characteristics (e.g., baseline hematologic counts, BMI), and bone marrow radiation dose-volume metrics (e.g., pelvic marrow volume receiving ≥20 Gy) (5–8). Despite these correlations, the predictive performance remains modest due to patient heterogeneity and complexity of marrow radiosensitivity (5). Consequently, individual risk prediction remains challenging, highlighting the need for more precise and personalized predictive biomarkers.

Radiomics, an emerging approach that extracts quantitative imaging features from routine medical images, offers promise for improving toxicity prediction (9, 10). Initial efforts utilizing computed tomography (CT)-based radiomics have demonstrated predictive value for hematologic toxicities in pelvic radiotherapy (11, 12). However, CT images primarily reflect bone density and trabecular structure, providing indirect and limited insight into marrow composition or functional status. Prior studies indicate that CT-derived radiomic features alone may not robustly capture marrow radiosensitivity due to their inability to differentiate hematopoietically active marrow from fatty marrow (12, 13).

Magnetic resonance imaging (MRI), with its superior soft-tissue contrast and capability to quantify marrow fat content and cellularity, provides a stronger rationale for radiomics-based marrow characterization. MRI can reliably distinguish active (red) marrow from inactive (fatty) marrow (14, 15). These findings highlight the potential of MRI-based radiomics to capture clinically relevant information about marrow status that is not discernible on CT. To date, however, no study has employed pre-treatment MRI-based radiomics to predict chemoradiotherapy hematologic toxicity; most existing models rely solely on CT images.

Given these considerations, this study aims to develop and validate a combined predictive model integrating clinical parameters and MRI-derived radiomics for predicting acute hematologic toxicity in patients undergoing pelvic radiotherapy for gynecologic cancers and other pelvic tumors. We hypothesize that combining clinical factors with MRI radiomics will significantly improve the accuracy and specificity of HT risk stratification, with the ultimate aim of enabling proactive identification of high-risk patients and informing bone marrow–sparing or supportive strategies in pelvic radiotherapy.

## 2 Materials and methods

### 2.1 Data collection

**Figure 1:**
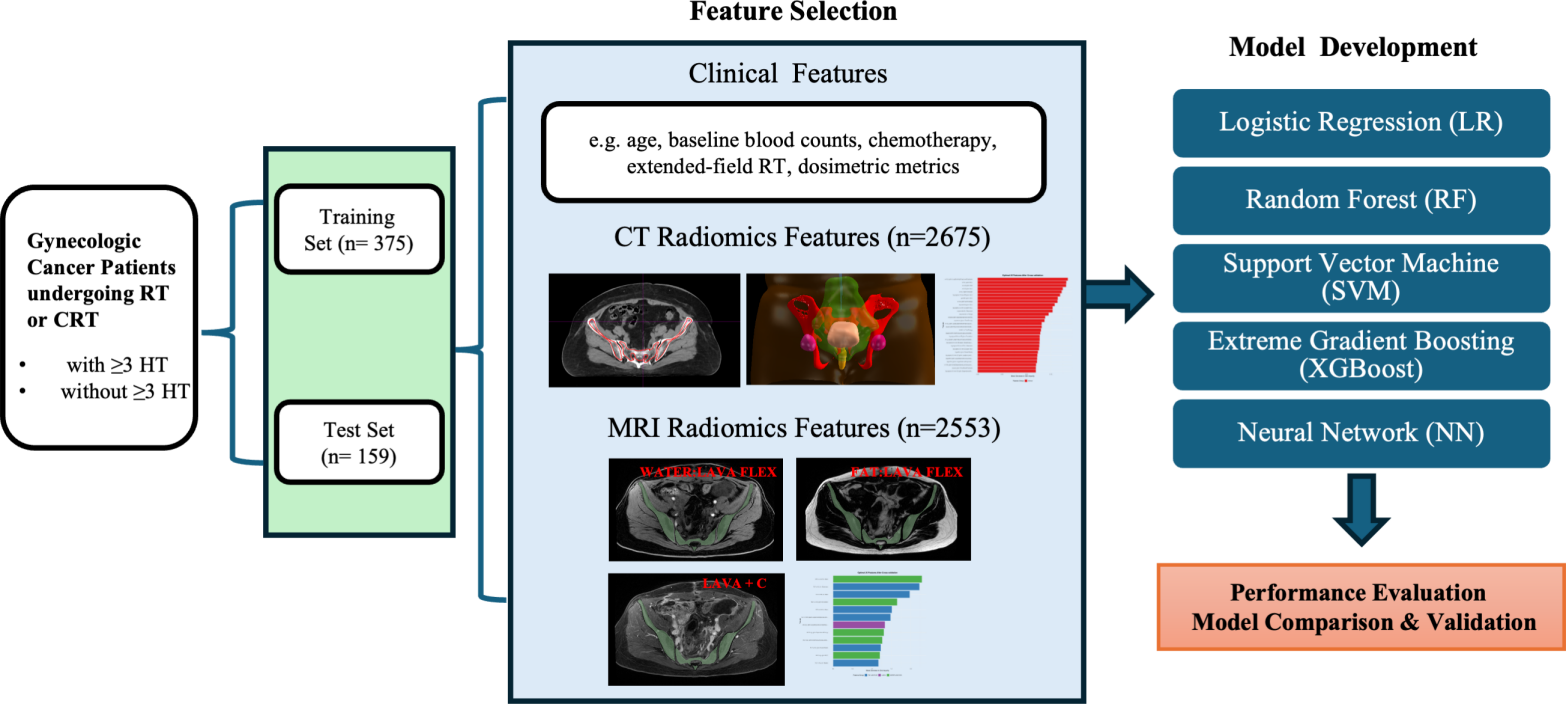
Workflow charts. HT, hematologic toxicity; RT, Radiotherapy; CRT, Chemoradiotherapy.

This study retrospectively analyzed 534 patients treated at Women’s Hospital, School of Medicine, Zhejiang University, between April 2023 and February 2025 for cervical or endometrial cancer with pelvic radiotherapy (RT). The requirement for informed consent was waived by the Ethics Committee of the Women’s Hospital, School of Medicine, Zhejiang University because this was a retrospective study using de-identified data. Eligible patients included: (1) cervical cancer patients receiving definitive radiotherapy, cervical or endometrial cancer receiving postoperative adjuvant radiotherapy; (2) treated with IMRT to the pelvic field (with or without extended-field to para-aortic nodes); (3) had weekly complete blood count from one week before RT through the end of RT; and (4) underwent pelvic contrast-enhanced magnetic resonance imaging (CE-MRI) at 3.0 Tesla and planning CT imaging before RT for radiomics analysis. Patients were excluded if they had prior pelvic radiation or extensive chemotherapy, pre-existing hematologic disorders, or significant bone lesions (e.g. pelvic bone metastases or replacement) that could affect marrow function. A summary of the study design is presented in **Figure 1**.

### 2.2 CT simulation and radiotherapy planning procedures

CT simulation and radiotherapy were performed with patients in the supine position and with a relatively full bladder. CT images covered the region extending from the lower lumbar spine to the entire pelvic cavity and were reconstructed using a Philips Brilliance Big Bore CT scanner system (Philips Healthcare, Best, the Netherlands) with a matrix size of 512 × 512 and a slice thickness of 5 mm. Clinical target volumes (CTVs) were delineated manually by experienced radiation oncologists according to guidelines established by the Radiation Therapy Oncology Group (RTOG). Pelvic bone marrow was delineated and included among the organs at risk (OARs) for external beam radiation therapy (EBRT) planning. EBRT planning and structural delineation were performed using the Pinnacle Treatment Planning System (Version 9.16.2, Philips Corp., Fitchburg, WI, USA). All manual contours were subsequently reviewed and validated by senior radiation oncologists specialized in gynecologic oncology to ensure standardization. EBRT was delivered utilizing IMRT on Elekta Infinity medical linear accelerator (Elekta, Stockholm, Sweden). Planning CT scan (non-contrast CT images acquired during CT simulation) was used for feature extraction.

### 2.3 MRI

All patients underwent a pelvic MRI scan (diagnostic MRI prior to RT). MRI was performed using a GE SIGNA Premier 3.0T magnetic resonance imaging system(General Electric Company HealthCare, USA)with gadobutrol as the contrast agent for enhanced imaging. Three sequences that highlighted bone marrow composition were acquired: (1) Axial contrast-enhanced Liver Acquisition with Volume Acceleration (Axial LAVA+C), repetition time (TR)=3.9 ms, echo time (TE)=1.7 ms, slice thickness=5.6 mm; (2) Axial Water LAVA-Flex; (3) Axial Fat LAVA-Flex, TR=6.1ms, TE =2ms, slice thickness=5.6mm.

### 2.4 Radiomics feature extraction (MRI and CT)

For radiomics analysis, we defined the region of interest (ROI) as the pelvic bone marrow within the radiation field. The pelvic bone marrow ROI was delineated on the planning CT and MRI, encompassing the internal volumes of the pelvic bones (sacrum, ilium, ischium, pubis, proximal femora) from the pelvic brim to the ischial tuberosities.

Radiomic feature extraction was performed using 3D Slicer (version 5.8.1) with the PyRadiomics extension (16). Prior to feature extraction, voxel intensity discretization was performed using a fixed bin width (25.0). For CT images, spatial filters including Haar wavelet decomposition and Laplacian of Gaussian (LoG) were applied to enhance textural representation (17, 18). For MRI images, only Haar wavelet decomposition was used, due to the inclusion of three distinct MRI sequences per patient, which would otherwise have generated excessive feature dimensionality. Finally, from CT images, 2,675 radiomic features were extracted per patient, and from MRI images, 2,553 radiomic features (851 features per MRI sequence) were obtained. These radiomic features comprised shape features (volumetric indices), first-order intensity statistics, and texture features computed from Gray-Level Co-occurrence Matrix (GLCM), Gray-Level Run-Length Matrix (GLRLM), Gray-Level Size Zone Matrix (GLSZM), Gray-Level Dependence Matrix (GLDM), and Neighboring Gray-Tone Difference Matrix (NGTDM) (19).

### 2.5 Hematologic toxicity assessment

All patients had baseline blood counts within one week prior to RT and weekly during treatment up to completion. We focused on four hematologic parameters: white blood cell count (leukocytes), absolute neutrophil count (ANC), hemoglobin, and platelet count. Acute hematologic toxicity (HT) was graded according to CTCAE v5.0 criteria (20) for leukopenia, neutropenia, anemia, and thrombocytopenia, using the nadir values during RT or within 4 weeks post-RT. For each patient, we determined the maximum grade of toxicity in any of the four categories. We defined “severe hematologic toxicity” as grade ≥3 toxicity (i.e. grade 3 or 4 in at least one parameter). This severe HT endpoint (yes/no) was used for predictive modeling.

### 2.6 Feature selection

Prior to feature selection, all continuous numerical features (including clinical and radiomic) were standardized using z-score normalization to ensure consistent feature scales. Features with near-zero variance or high inter-feature correlation (Pearson’s r > 0.9) were removed. Given the large dimensionality of radiomic features relative to the sample size, a two-step feature selection approach was used. Two supervised feature selection methods were used: (1) LASSO logistic regression, a regularization technique that imposes an L1 penalty to promote sparsity in feature selection, tuned via cross-validation (21); (2) Random Forest feature importance ranking using mean decrease in Gini impurity (18). The final feature set was determined by combining non-zero LASSO features and the top 30 Random Forest features, followed by cross-validation to optimize the number of features included in each model.

### 2.7 Model development

All models were trained using 5-fold cross-validation (k = 5), with AUC used as the primary evaluation metric during feature selection and hyperparameter tuning. Five classifiers were trained and evaluated using the selected features: 1) Logistic Regression (LR): a generalized linear model used for binary classification (22). 2) Random Forest (RF): an ensemble classifier using bootstrapped decision trees (18). 3) Support Vector Machine (SVM): implemented with a radial basis function (RBF) kernel and cross-validated tuning (23). 4) Extreme Gradient Boosting (XGBoost): a tree-based boosting algorithm optimized with hyperparameter tuning (24). 5)Neural Network (NN): a multi-layer perceptron with one hidden layer and L2 regularization (25). Performance was evaluated on the test set using area under the ROC curve (AUC), accuracy, sensitivity, specificity, and F1-score.

Finally, the tuned models were applied to the unseen test set (n=159) to evaluate performance. We calculated the AUC for predicting severe HT in the test set for each classifier, as well as accuracy, sensitivity (recall of severe HT cases), specificity, and F1-score. The primary metric of interest was AUC.

All model development was conducted in R (v4.4) and using packages glmnet, randomForest, e1071, xgboost, caret, pROC, ggplot2, MASS, rpart and tidyr.

## 3 Results

### 3.1 Patient characteristics

Of the 534 patients, 480 had cervical cancer (380 received postoperative RT after hysterectomy; 100 received definitive RT), and 54 had endometrial cancer (postoperative RT). All patients were treated with EBRT to the pelvis using IMRT (typically 45–50.4 Gy in 25–28 fractions). A subset (17% in training, 21% in test) also received prophylactic para-aortic nodal irradiation (extended-field IMRT). The training (n=375) and test (n=159) sets were well-balanced in clinical characteristics (**Table 1**). Concurrent chemotherapy (weekly cisplatin, 40 mg/m²) was administered in 68% of patients (65.1% of training cohort vs 73.6% of test cohort; p=0.068), primarily those with cervical cancer. After EBRT, intracavitary brachytherapy was delivered for definitive cervical cancer cases and postoperative cervical cancer cases with positive vaginal surgical margins (32.4% of training, 30.8% of test patients; p=0.435). Baseline patient characteristics were comparable between the training (n=375) and independent test set (n=159), with no significant differences in age (median ~58 years), disease type distribution, doses to bone marrow, baseline blood counts and plasma protein level (all p>0.05).

**Table 1:** Clinical patient characteristics (Training set vs. Test set).

Variable	Training Set(n=375)	Test Set (n=159)	P value
Disease type			0.532
Postoperative cervical cancer	272 (72.5%)	108 (67.9%)	
Cervical cancer	66 (17.6%)	34 (21.4%)	
Postoperative uterine tumor	37 (9.9%)	17 (10.7%)	
Age (years)			0.218
(median, mean ± SD, range)	58, 57.6 ± 12.1, 24-88	57, 56.1 ± 12.8, 27-84	
CDDP concurrent chemotherapy			0.068
Yes	244 (65.1%)	117 (73.6%)	
No	131 (34.9%)	42 (26.4%)	
Para-aortic extended-field			0.166
Yes	58 (15.5%)	33 (20.8%)	
No	317 (84.5%)	126 (79.2%)	
Brachytherapy			0.435
Yes:	121 (32.4%)	49 (30.8%)	
No	254 (67.7%);	110 (69.2%);	
Acute hematotoxicity			1.000
≥3	100 (26.7%)	42 (26.4%)	
<3	275 (73.3%)	117 (73.6%)	
Doses to bone marrow (median, mean ± SD, range)			
Maximum dose (Gray)	51.8, 53.8 ± 5.3, 30.6-66.3	2.9, 54.4± 4.8, 43.2-64.9	0.208
Mean dose (Gray)	31.2, 31.0 ± 2.0, 14.1-33.8	31.1, 31.1 ± 1.4, 23.8-33.7	0.927
Minimum dose (Gray)	5.8, 5.5 ± 1.5, 1.2-10.8	5.7, 5.4 ± 1.4, 1.9-8.0	0.946
Volume receiving 25 Gray (cm^3^)	195.89, 198.05 ± 35.26, 115.44-302.13	189.59, 188.82 ± 30.68, 122.07-264.61	0.186
Volume receiving 25 Gray (%)	69.61, 68.96 ± 4.72, 58.15-79.55	70.76, 69.62 ± 5.64, 48.23-79.10	0.169
Baseline (median, mean ± SD, range)			
White blood cell count (10^9/L)	5.6, 6.4 ± 1.9, 2.7-12.9	5.8, 5.9 ± 1.74, 2.8-15.6	0.982
Hemoglobin (g/L)	120.0, 118.8 ± 13.0, 73.0-150.0	119.0, 116.7 ± 14.6, 31.0-163.0	0.133
Platelet (10^9/L)	245.0, 253.2 ± 77.0, 44.0-552.0	235.0, 247.0 ± 78.4, 102.0-900.0	0.196
Albumin (g/L)	41.6, 41.3 ± 2.9, 31.1-48.6	41.7, 41.4 ± 3.1, 31.3-50.1	0.784
Total protein (g/L)	71.0, 70.6 ± 4.9, 55.6-84.6	71.3, 71.0 ± 5.0, 55.9-87.5	0.594

In the entire cohort, 259 patients (48.5%) experienced at least one Grade 2 hematologic toxicity during RT, and 142 patients (26.6%) experienced Grade ≥3 (severe) hematologic toxicity. The distribution of HT grades in each subset is detailed in **Table 2**. Leukopenia was the most common toxicity: over 68% of patients had developed acute leukopenia (grade ≥2), with a few grade 4 cases in training set. Neutropenia often accompanied leukopenia; around 8% had grade 3 or 4 neutropenia. Anemia was frequently observed at low grades (over one-third had grade 1), but only ~5% had grade 3 anemia (1% grade 4). Thrombocytopenia was mild in most cases (grade 0–1 in >90%); grade 3 thrombocytopenia occurred in ~2–3% of patients. Overall, only ~6–8% of patients had no hematologic toxicity at all (grade 0 for all indices), reflecting that the vast majority experienced at least transient cytopenias during pelvic RT. 

**Table 2:** Hematologic toxicity summary.

	Hematologic toxicity	Grade 0 (n, %)	Grade 1 (n, %)	Grade 2 (n, %)	Grade 3 (n, %)	Grade 4 (n, %)
Train set	Leukopenia	43 (11.5%)	75 (20.0%)	179 (47.7%)	74 (19.7%)	4 (1.1%)
	Anemia	127 (33.9%)	139 (37.1%)	85 (22.7%)	19 (5.1%)	5 (1.3%)
	Thrombocytopenia	271 (72.3%)	66 (17.6%)	27 (7.2%)	10 (2.7%)	1 (0.3%)
	Neutropenia	150 (40.0%)	109 (29.1%)	86 (22.9%)	25 (6.7%)	5 (1.3%)
	Hematotoxicity	29 (7.7%)	66 (17.6%)	180 (48.0%)	90 (24.0%)	10 (2.7%)
Test set	Leukopenia	16 (10.1%)	32 (20.1%)	74 (46.5%)	37 (23.3%)	0 (0.0%)
	Anemia	58 (36.5%)	52 (32.7%)	41 (25.8%)	7 (4.4%)	1 (0.6%)
	Thrombocytopenia	114 (71.7%)	30 (18.9%)	10 (6.3%)	4 (2.5%)	1 (0.6%)
	Neutropenia	62 (39.0%)	49 (30.8%)	34 (21.4%)	14 (8.8%)	0 (0.0%)
	Hematotoxicity	8 (5.0%)	30 (18.9%)	79 (49.7%)	40 (25.2%)	2 (1.3%)

### 3.2 Clinical predictors of hematologic toxicity

**Figure 2:**
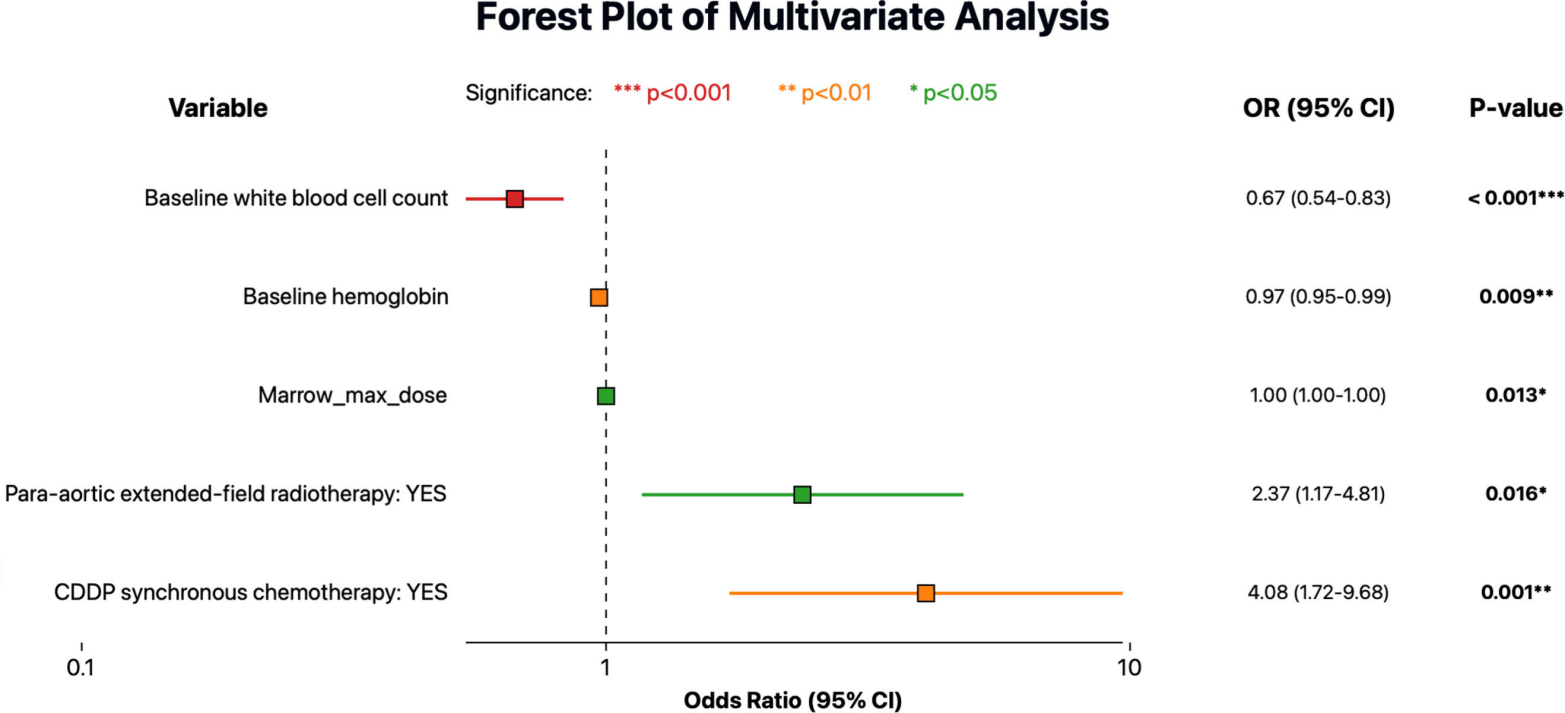
Forest plot showing multivariate logistic regression analysis for predictors of severe acute hematologic toxicity (HT). Odds ratios (OR) are indicated by colored squares according to statistical significance levels, and horizontal lines represent the 95% confidence intervals (CI).

On univariate analysis, several clinical factors were associated with increased HT: concurrent chemotherapy, larger irradiated volume (para-aortic extended-field), lower baseline white blood cell count/hemoglobin, and higher Bone marrow dose metrics. In the multivariate logistic regression (**Figure 2**), five factors remained independently significant. Concurrent chemotherapy had the strongest association with severe HT (OR ~4, *p*=0.001). Baseline bone marrow function (as reflected by baseline white blood cell count and hemoglobin) was also critical, highlighting that patients with stronger baseline counts better tolerated radiotherapy without severe cytopenias. As expected, para-aortic RT was associated with higher HT risk in our data (OR ~2.37, *p*<0.05). Finally, a higher maximum bone marrow dose predicted higher HT risk (OR ~1.003, p<0.05). While the effect per unit was small, it was statistically significant, reinforcing that avoiding very high point doses to active marrow (hotspots, e.g. in lumbosacral bone) could be beneficial.

### 3.3 Clinical model performance

**Figure 3:**
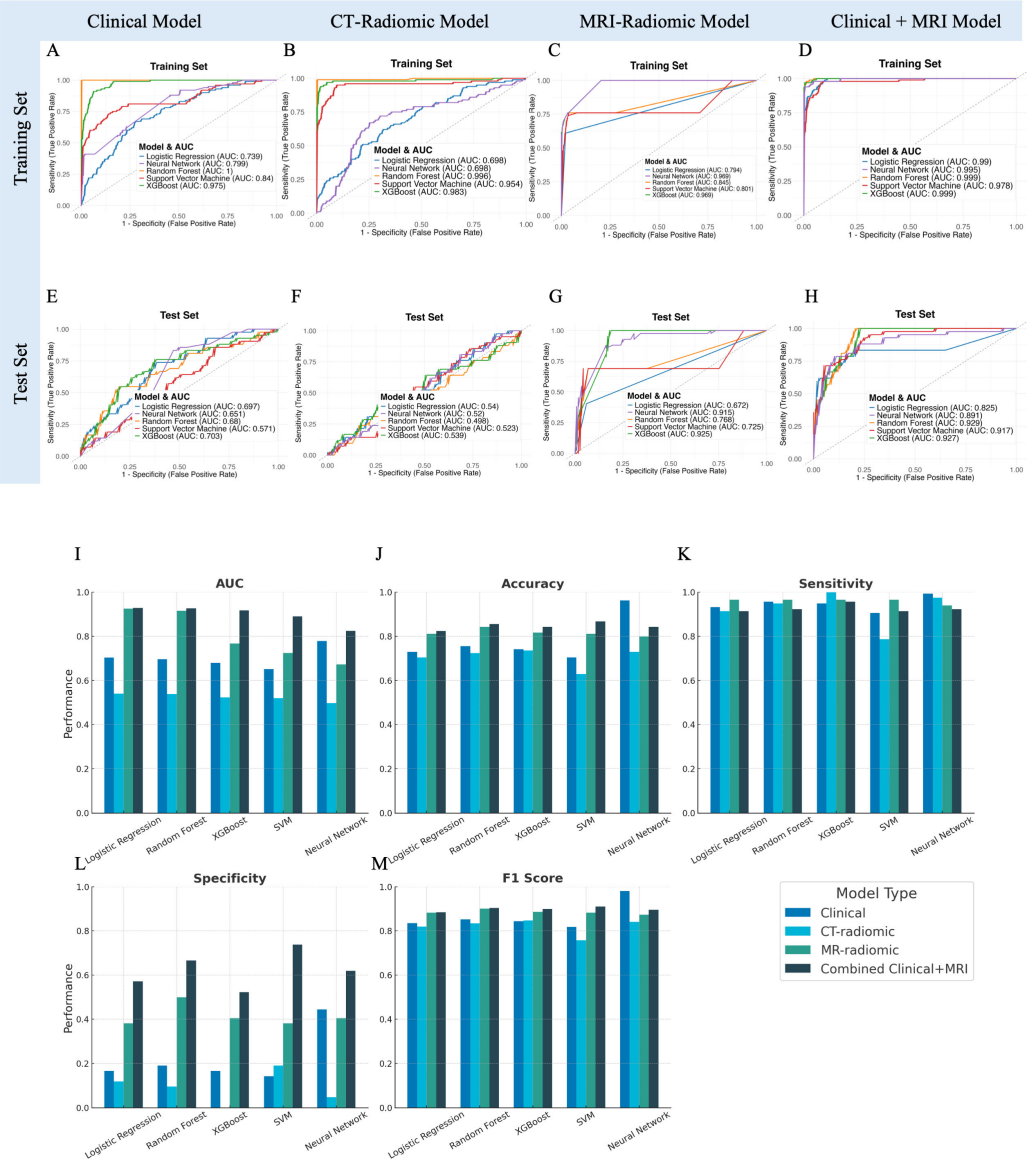
Performance evaluation and comparison of predictive models for acute hematologic toxicity using clinical factors, CT radiomics, MRI radiomics, and combined clinical-MRI radiomics features. **(A–D)** Receiver operating characteristic (ROC) curves illustrating model performance on the training set. **(E–H)** ROC curves for the independent test set. **(I–M)** Bar charts comparing performance metrics (AUC, Accuracy, Sensitivity, Specificity, F1 Score) across the four modeling strategies in the test dataset.

Model performance on the independent test set was notably poorer than in the training set. The clinical models showed suboptimal generalizability, with test set AUCs ranging from 0.57 to 0.70 and notably low specificity (<20%) despite high sensitivity (>90%) (**Figure 3A, E**, **Supplementary Table 1**). This discrepancy highlights considerable overfitting: although clinical features especially chemotherapy status allowed models to correctly identify nearly all patients who developed severe hematologic toxicity, they also excessively flagged patients as high-risk, resulting in numerous false-positive predictions and low practical specificity despite excellent initial training performance.

### 3.4 CT-radiomics model performance

The predictive performances of the five machine learning models using the top 30 selected CT-based radiomic features are presented in **Figure 3B, F**, **Supplementary Table 1**. On the independent test set, accuracy ranged from approximately 63% (neural network) to 74% (SVM), with modest AUC values (0.50–0.54). Although sensitivity was high, specificity was uniformly low (0–19%), indicating frequent false-positive predictions. These results suggest that, despite rigorous feature selection, CT-based radiomic features alone provided limited predictive value for acute hematologic toxicity. The selected features were predominantly texture- and intensity-based metrics extracted from filtered images (e.g., wavelet, exponential, gradient), reflecting marrow heterogeneity that was insufficient to support robust prediction compared to MRI radiomics.

### 3.5 MRI-radiomics model performance

We next evaluated the predictive performance of models based on MRI radiomic features of the bone marrow. A total of 50 MRI-based radiomic features were selected by our feature selection process to feed into the classifiers. These included features primarily included first-order intensity (mean, median, skewness) and advanced texture metrics (e.g., GLCM correlation, GLSZM Large Area High Gray Level Emphasis, GLDM entropy and uniformity measures). On the independent test set, models achieved good discrimination, notably the XGBoost (AUC=0.925, accuracy=81.1%) and neural network models (AUC=0.915, accuracy=84.3%). These models exhibited high sensitivity (~97%) and moderate specificity (38–50%), indicating effective identification of patients at high risk of severe hematologic toxicity (**Figures 3C, G**, **Supplementary Table 1**). In conclusion, MRI features likely reflect marrow composition and heterogeneity, contributing to their superior predictive power compared to CT-radiomics.

### 3.6 Combined clinical and MRI-radiomics model performance

**Figure 4:**
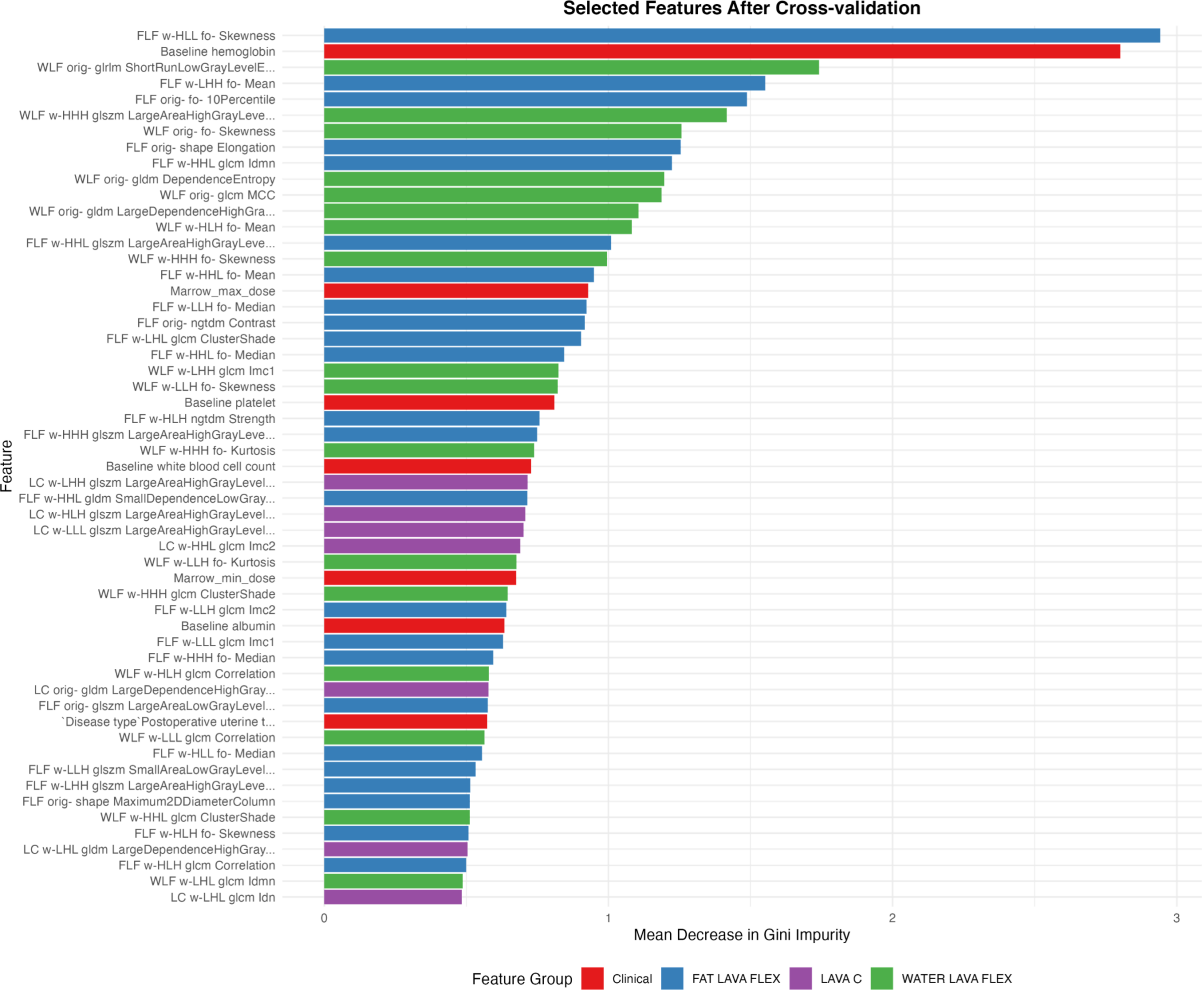
Radiomic and clinical features selected after cross-validation ranked by their relative importance. Features are color-coded according to their source: clinical parameters (red), and MRI-derived radiomic features from Fat LAVA-Flex (blue), Water LAVA-Flex (green), and LAVA+C (purple) imaging sequences.

Given that both clinical factors and MRI radiomic features demonstrated strong predictive value individually, we developed a combined model integrating the features from both domains. The combined model integrated a total of 55 rigorously selected features, encompassing both key clinical parameters and MRI-derived radiomic metrics (**Figure 4**). The clinical features primarily included baseline hematologic, radiation dosage metrics and disease type. MRI-radiomic features primarily included intensity and texture metrics derived from three sequences (LAVA+C, Water LAVA-Flex, and Fat LAVA-Flex), encompassing first-order statistics (e.g., mean, median, skewness, kurtosis, 10 Percentile), texture features (GLCM, GLSZM, GLDM, NGTDM) and shape metrics.

On the test set, this combined model outperformed all single-modality models. The XGBoost classifier achieved the highest AUC (0.927), with accuracy of 85.5%, sensitivity of 92.3%, and specificity of 66.7%. The neural network also performed well (AUC = 0.891, accuracy = 86.8%, specificity = 73.8%). Random Forest and SVM models yielded comparable AUCs (0.929 and 0.917), while logistic regression showed slightly lower specificity (61.9%).

**Figures 3I–M** compares the predictive performance of clinical, CT-radiomics, MRI-radiomics, and combined models. The combined model consistently achieved the best performance, with improved accuracy (up to 86.8%) and notably higher specificity (up to 73.8%) compared to individual models. Although sensitivity remained high across all models, the combined model’s enhanced specificity suggests more precise risk stratification and reduced false positives.

Overall, integrating clinical data with MRI-derived radiomics markedly improved prediction accuracy, specificity, and overall discriminative power compared to either clinical or imaging features alone.

### 3.7 Subtype-specific predictive performance of the combined clinical and MRI-radiomic model

**Figure 5:**
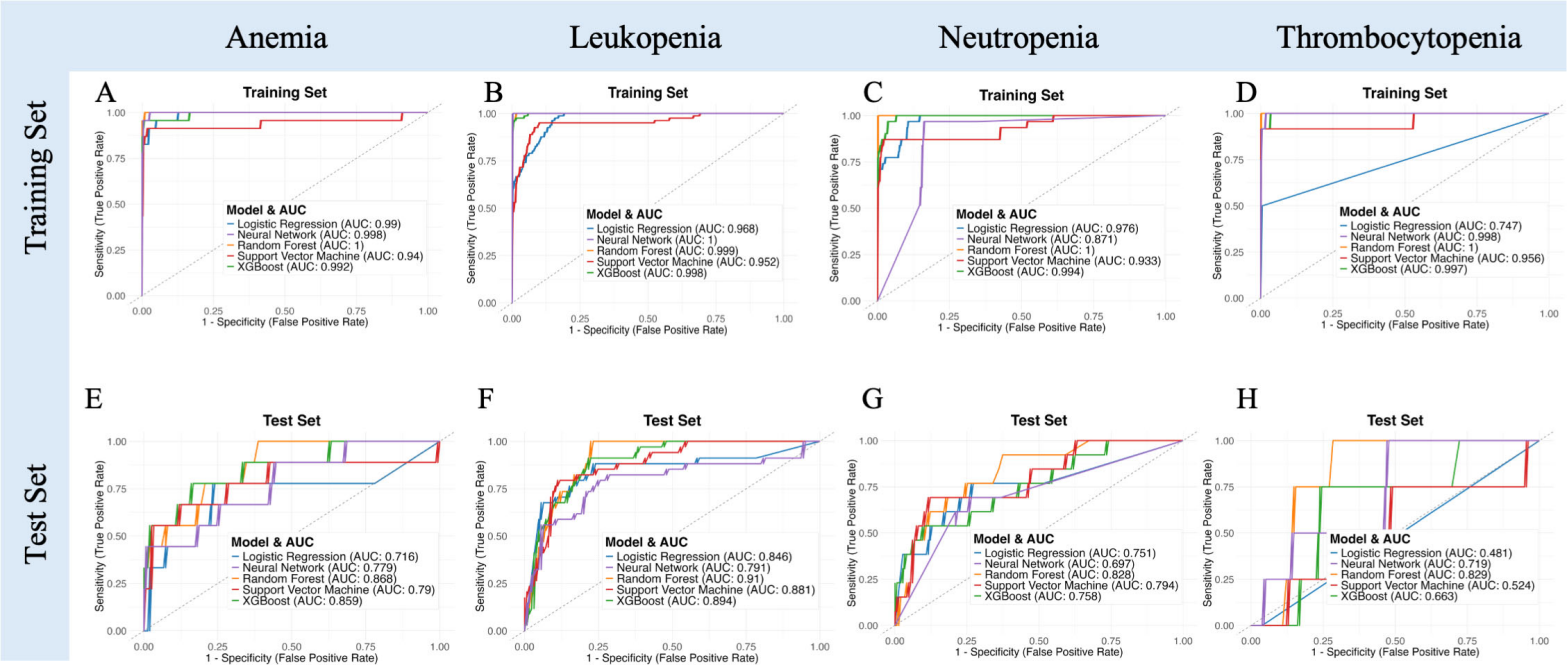
Receiver operating characteristic (ROC) curves illustrating model performance in predicting specific subtypes of severe acute hematologic toxicity (anemia, leukopenia, neutropenia, thrombocytopenia) for both training **(A–D)** and independent test sets **(E–H)**.

The predictive performances of the combined clinical and MRI-radiomic model for identifying severe (Grade ≥3) hematologic toxicities across four different subtypes (anemia, leukopenia, neutropenia, and thrombocytopenia) are summarized in **Figure 5**, **Supplementary Table 2**. Among these hematologic toxicities, the predictive accuracy varied notably. Random Forest achieved the highest AUCs for anemia (0.868), leukopenia (0.910), and neutropenia (0.828). However, prediction for thrombocytopenia was limited by severe class imbalance (positive:negative ≈ 1:32), resulting in low specificity (0.0) despite a moderate AUC (0.829), reflecting difficulty in correctly identifying negative cases. This was likely influenced by the class imbalance. Thrombocytopenia was the most severely imbalanced (positive: negative ratio approximately 1:32), which significantly limited the model’s ability to reliably predict true negatives, resulting in notably lower specificity and overall predictive stability.

### 3.8 Predictive performance of the combined clinical and MRI-radiomic model stratified by treatment modality

**Figure 6:**
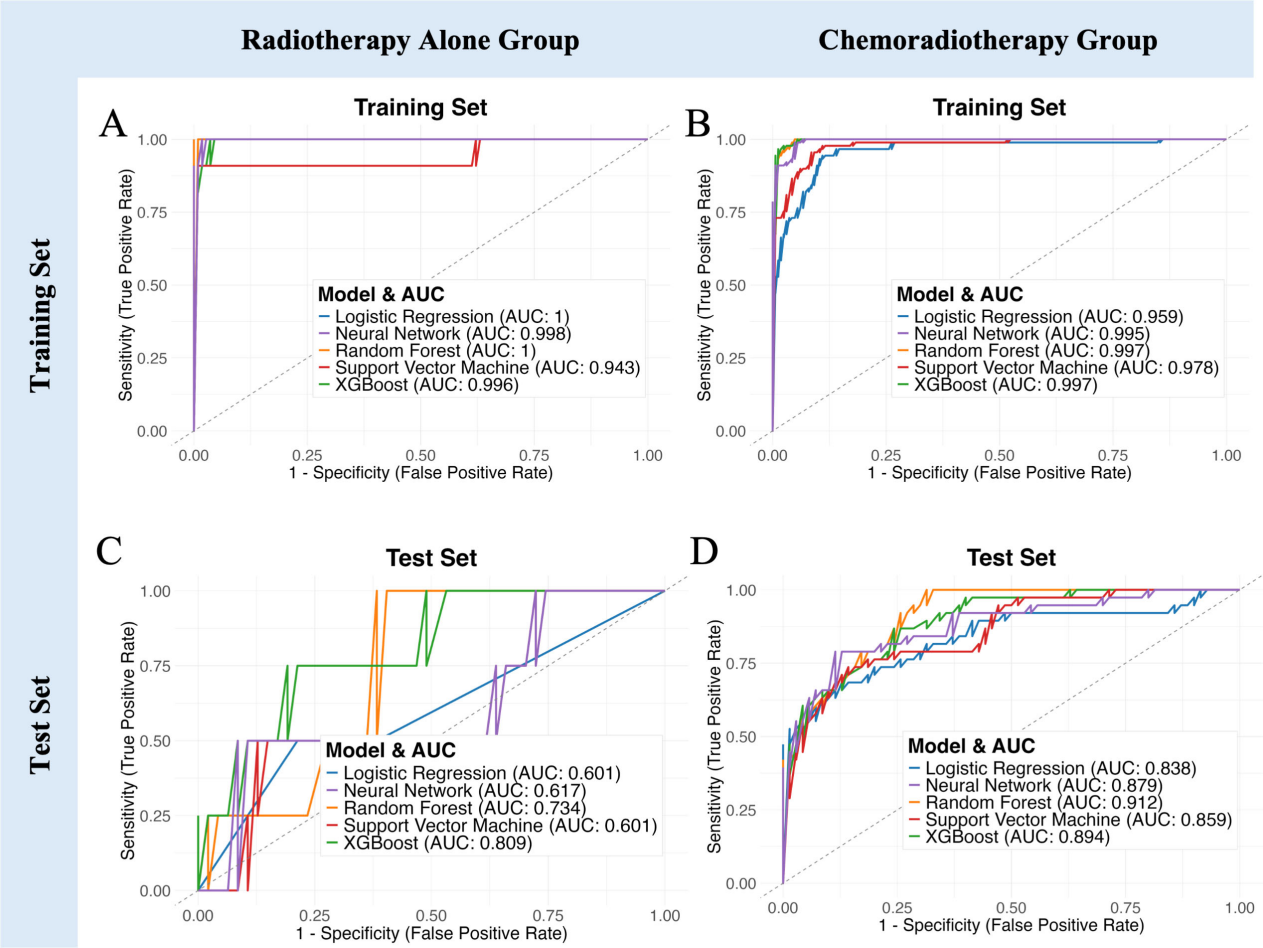
Model performance comparison stratified by treatment regimen. **(A, B)** ROC curves demonstrating predictive capability of five machine learning models in the training set for Radiotherapy Alone and Chemoradiotherapy groups, respectively. **(C, D)** ROC curves for the independent test set. **(E–I)** Bar charts showing detailed performance metrics (AUC, Accuracy, Sensitivity, Specificity, F1 Score) across Radiotherapy Alone group, Chemoradiotherapy group, and all patients group.

The combined clinical and MRI-radiomic model exhibited treatment-dependent performance differences (**Figure 6**, **Supplementary Table 3**). In the test set, predictive accuracy was higher in the CRT subgroup (AUC up to 0.912) than in the RT-alone subgroup (AUC up to 0.809). Although RT-alone models showed high accuracy, specificity was consistently low (≤0.25), suggesting overfitting. In contrast, the CRT group achieved better balance between sensitivity and specificity, indicating robust discriminative capacity. These results highlight treatment-dependent differences in model performance, emphasizing that the combined MRI radiomics and clinical model is particularly beneficial for predicting hematologic toxicity risk in patients receiving concurrent chemoradiotherapy.

## 4 Discussion

In this study, we developed predictive models for acute hematologic toxicity (AHT) in gynecologic cancer patients receiving pelvic radiotherapy, leveraging both clinical factors and radiomic features of the pelvic bone marrow. Our MRI‐based radiomic model demonstrated superior predictive performance for AHT compared to the CT‐based model. For comparison, Le et al. reported that a CT radiomics model combined with clinical features achieved an AUC of approximately 0.80–0.85 in predicting severe AHT (11). In our study, further integrating MRI radiomic features with clinical parameters led to the highest overall accuracy among all models, highlighting the added value of MRI radiomic features.

These findings highlight the value of MRI in capturing biologically relevant information that CT cannot provide. MRI offers better soft-tissue contrast and enables differentiation between active and fatty marrow through specialized sequences (e.g., LAVA+C, Water and Fat LAVA-Flex), which together provide detailed information on marrow composition, vascularity, and fat content. Radiomic features extracted from these sequences—such as intensity statistics and texture metrics—capture spatial heterogeneity and microstructural variations within the marrow, reflecting its hematopoietic capacity more precisely than CT-based density features (13–15, 26). Prior studies support this advantage: Carmona et al. showed that MRI fat fraction quantification is highly sensitive to bone marrow compositional changes during chemoradiotherapy and that these changes correlate strongly with declines in peripheral blood counts (13). While these MRI sequences can quantify marrow fat content directly, radiomic analysis adds value by capturing spatial heterogeneity, texture, and signal distribution within the marrow. Instead of relying solely on global fat fraction values, radiomic features—including first-order statistics (e.g., entropy, uniformity) and higher-order texture metrics (e.g., GLCM, GLRLM)—detect subtle microregional variations in marrow architecture that may reflect differential hematopoietic capacity. This complementary information enables a more nuanced assessment of radiosensitivity and improves predictive modeling. Prior studies support this advantage: Qin et al. demonstrated that functional MRI radiomic features can detect microscopic marrow changes across various dose levels, providing an objective basis for bone-marrow sparing strategies (15). In contrast, CT-based radiomics primarily capture density and structural variations that do not directly reflect bone marrow reserve or function. Thus, MRI’s ability to probe the marrow’s fat content and microarchitecture gives it a biologic edge in predicting hematologic toxicity. Notably, our feature selection identified several MRI-derived texture and intensity features (from fat and water images) related to marrow fat distribution, supporting the notion that the spatial arrangement of active marrow is a critical predictor of hematologic tolerance.

Our findings also concord with the emerging evidence that MRI-defined active bone marrow is the key subvolume driving hematologic toxicity. For example, Ke et al. delineated “red marrow” on pelvic MRI and found that higher radiation doses to this active marrow (such as bone marrow V15) were significantly associated with increased risk of acute HT (27). This suggests that MRI can localize the functionally important marrow regions that should be preferentially spared. In our study, even without explicit sparing, the MRI radiomic model may inherently focus on these active regions (through its features), explaining its stronger predictive power. Together, these considerations provide a compelling technical and biological rationale for why the MRI-based model outperforms the CT-based model in forecasting hematologic toxicity.

The predictive utility of our models was more pronounced in patients receiving CRT than in those undergoing RT alone. The MRI model maintained robust performance in the CRT subgroup, correctly stratifying high-risk patients, whereas prediction in the RT-alone subgroup was more modest. This trend aligns with recent findings from a large cervical cancer cohort, where AHT risk models performed significantly better in CRT patients than in RT-only patients (28). Since MRI scans were acquired prior to treatment, the radiomic features likely reflect preexisting differences in bone marrow reserve or function. These baseline differences may have a greater clinical impact in patients receiving CRT, where the hematologic stress is more severe, thereby amplifying the consequences of underlying marrow vulnerability. In contrast, RT alone exerts milder marrow suppression, and preexisting differences may not translate into significant toxicity. This may explain the stronger predictive power observed in the CRT subgroup, and underscores the potential of MRI radiomics as a pre-treatment biomarker of marrow resilience under intensive therapy.

The superior performance of the predict model has practical significance. Early identification of patients at high risk for severe hematologic toxicity could enable personalized interventions to maintain treatment intensity. For instance, patients flagged by the combined model as high-risk might benefit from prophylactic measures such as bone marrow–sparing radiation techniques, dose reduction of concurrent chemotherapy, or growth factor support (e.g., Granulocyte Colony-Stimulating Factor), in order to prevent interruptions in therapy. AHT is known to compromise treatment efficacy and prolong the overall treatment time (29); therefore, a reliable prediction model is clinically valuable to trigger timely supportive care. Our combined model – which incorporated MRI radiomic features along with key clinical factors (notably baseline hemoglobin and other blood counts) – achieved the highest discrimination. This reinforces that a multifactorial approach is optimal: integrating radiomic signatures with clinical variables improves predictive accuracy relative to radiomics or clinical data alone (26). In practice, such a model could be deployed as a nomogram or decision tool before treatment to stratify patients by toxicity risk. High-risk patients could then be closely monitored and managed proactively, whereas low-risk patients may proceed with standard treatment protocols. Ultimately, this strategy could reduce unplanned dose delays and ensure patients complete chemoradiation on schedule, potentially improving oncologic outcomes.

We acknowledge several limitations in our study. First, the sample size is moderate and drawn from a single institution; larger multi-center validations are warranted to ensure the model’s generalizability. Radiomics models can be prone to overfitting, especially with high-dimensional feature spaces – we mitigated this via feature selection and cross-validation, but external confirmation remains essential. Subtype prediction, particularly for rare toxicities like grade ≥3 thrombocytopenia, was challenged by severe class imbalance and limited events, affecting specificity. Larger, balanced datasets and rebalancing strategies are needed for improved modeling of these subgroups. Additionally, our models did not explicitly include advanced dose metrics beyond the basic dose-volume variables – incorporating “dosiomic” features (radiomics of the 3D dose distribution) alongside imaging features is an intriguing approach that could further improve prediction.

Going forward, prospective trials should be considered to test whether acting on model predictions can indeed reduce hematologic toxicity. For example, a trial could stratify patients by a baseline MRI radiomics risk score and assign high-risk patients to an intensified supportive care or marrow-sparing plan. Such prospective validation would establish the clinical utility of our combined model beyond a retrospective proof-of-concept. In parallel, exploration of AI explainability methods might help illuminate which imaging phenotypes drive toxicity risk, enhancing clinician trust in the model’s predictions.

Our study demonstrates that a combined clinical and MRI-radiomic model accurately predicts acute hematologic toxicity in patients undergoing pelvic radiotherapy. MRI radiomics outperformed CT-based models, especially in chemoradiotherapy-treated patients, highlighting MRI’s potential as a noninvasive imaging biomarker. Future validation and integration into personalized treatment planning may significantly reduce treatment-related hematologic complications.

## Data Availability

The raw data supporting the conclusions of this article will be made available by the authors, without undue reservation.
