# Supplementary material for: Combined clinical and MRI-based radiomics model for predicting acute hematologic toxicity in gynecologic cancer radiotherapy
Source: Front Oncol. 2025 Aug 8;15:1644053. doi: 10.3389/fonc.2025.1644053 (PMC12370453; doi:10.3389/fonc.2025.1644053)
Supplement: Supplementary file 1 [file Table1.docx]

**Supplementary Table 1. Performance of Clinical /CT-radiomic/MRI-radiomic/ Combined Models in predicting severe (Grade ≥3) hematologic toxicity.**

|  | **Model** | **Accuracy** | **AUC** | **Sensitivity** | **Specificity** | **F1 Score** |
| --- | --- | --- | --- | --- | --- | --- |
| **Clinical Model** | **XGBoost** | **0.7296** | **0.7033** | **0.9316** | **0.1667** | **0.8352** |
|  | Logistic Regression | 0.7547 | 0.6970 | 0.9573 | 0.1905 | 0.8517 |
|  | Random Forest | 0.7421 | 0.6798 | 0.9487 | 0.1667 | 0.8441 |
|  | Neural Network | 0.7044 | 0.6514 | 0.9060 | 0.1429 | 0.8185 |
|  | SVM | 0.7233 | 0.5708 | 0.9573 | 0.0714 | 0.8358 |
| **CT-radiomic Model** | **Logistic Regression** | **0.7044** | **0.5400** | **0.9145** | **0.1190** | **0.8199** |
|  | XGBoost | 0.7233 | 0.5392 | 0.9487 | 0.0952 | 0.8346 |
|  | SVM | 0.7358 | 0.5229 | 1.0000 | 0.0000 | 0.8478 |
|  | Neural Network | 0.6289 | 0.5198 | 0.7863 | 0.1905 | 0.7572 |
|  | Random Forest | 0.7296 | 0.4979 | 0.9744 | 0.0476 | 0.8413 |
| **MR-radiomic Models** | **XGBoost** | **0.8113** | **0.9249** | **0.9658** | **0.3810** | **0.8828** |
|  | Neural Network | 0.8428 | 0.9149 | 0.9658 | 0.5000 | 0.9004 |
|  | Random Forest | 0.8176 | 0.7679 | 0.9658 | 0.4048 | 0.8863 |
|  | SVM | 0.8113 | 0.7249 | 0.9658 | 0.3810 | 0.8828 |
|  | Logistic Regression | 0.7987 | 0.6725 | 0.9402 | 0.4048 | 0.8730 |
| **Combined Clinical and MRI-radiomics Model** | **Random Forest** | **0.8239** | **0.9287** | **0.9145** | **0.5714** | **0.8843** |
|  | XGBoost | 0.8553 | 0.9267 | 0.9231 | 0.6667 | 0.9038 |
|  | Neural Network | 0.8428 | 0.9170 | 0.9573 | 0.5238 | 0.8996 |
|  | SVM | 0.8679 | 0.8909 | 0.9145 | 0.7381 | 0.9106 |
|  | Logistic Regression | 0.8428 | 0.8251 | 0.9231 | 0.6190 | 0.8963 |

Results are shown for the test set (n=159), for five classifiers: Logistic Regression, Random Forest, Extreme Gradient Boosting (XGBoost), Support Vector Machine (SVM), and a Neural Network. Sensitivity and Specificity refer to the true positive rate and true negative rate, respectively, for identifying patients with the toxicity. Bold values highlight the highest AUC achieved for each subtype.

**Supplementary Table 2. Performance of combined model (Clinical and MRI-radiomic) in predicting severe (Grade ≥3) hematologic toxicity subtypes.**

|  | Model | Accuracy | AUC | Sensitivity | Specificity | F1 Score |
| --- | --- | --- | --- | --- | --- | --- |
| **Anemia** | Logistic Regression | 0.9371 | 0.7163 | 0.9733 | 0.3333 | 0.9669 |
|  | Random Forest | 0.956 | **0.8681** | 0.9933 | 0.3333 | 0.977 |
|  | XGBoost | 0.9623 | 0.8593 | 1 | 0.3333 | 0.9804 |
|  | SVM | 0.9434 | 0.7896 | 1 | 0 | 0.9709 |
|  | Neural Network | 0.9623 | 0.7785 | 0.9933 | 0.4444 | 0.9803 |
| **Leukopenia** | Logistic Regression | 0.8679 | 0.846 | 0.92 | 0.6765 | 0.9163 |
|  | Random Forest | 0.8428 | **0.9104** | 0.936 | 0.5 | 0.9035 |
|  | XGBoost | 0.8491 | 0.8941 | 0.936 | 0.5294 | 0.907 |
|  | SVM | 0.8302 | 0.8807 | 0.944 | 0.4118 | 0.8973 |
|  | Neural Network | 0.8113 | 0.7906 | 0.872 | 0.5882 | 0.879 |
| **Neutropenia** | Logistic Regression | 0.8813 | 0.7507 | 0.9252 | 0.3846 | 0.9347 |
|  | Random Forest | 0.9125 | **0.8276** | 0.9796 | 0.1538 | 0.9536 |
|  | XGBoost | 0.9313 | 0.7582 | 0.9932 | 0.2308 | 0.9637 |
|  | SVM | 0.9188 | 0.7938 | 1 | 0 | 0.9577 |
|  | Neural Network | 0.9188 | 0.6968 | 1 | 0 | 0.9577 |
| **Thrombocytopenia** | Logistic Regression | 0.9371 | 0.4806 | 0.9613 | 0 | 0.9675 |
|  | Random Forest | 0.9686 | **0.829** | 0.9935 | 0 | 0.984 |
|  | XGBoost | 0.9686 | 0.6629 | 0.9935 | 0 | 0.984 |
|  | SVM | 0.9748 | 0.5242 | 1 | 0 | 0.9873 |
|  | Neural Network | 0.9497 | 0.7194 | 0.9742 | 0 | 0.9742 |

Results are shown for the test set (n=159), for five classifiers: Logistic Regression, Random Forest, Extreme Gradient Boosting (XGBoost), Support Vector Machine (SVM), and a Neural Network. Sensitivity and Specificity refer to the true positive rate and true negative rate, respectively, for identifying patients with the toxicity. Bold values highlight the highest AUC achieved for each subtype.

**Supplementary Table 3. Performance of combined model (Clinical and MRI-radiomic) in predicting severe (Grade ≥3) hematologic toxicity in different treatment groups.**

|  | Model | Accuracy | AUC | Sensitivity | Specificity | F1 Score |
| --- | --- | --- | --- | --- | --- | --- |
| **Radiotherapy alone group** | XGBoost | 0.9020 | 0.8085 | 0.9574 | 0.2500 | 0.9474 |
|  | Random Forest | 0.9020 | 0.7340 | 0.9574 | 0.2500 | 0.9474 |
|  | Neural Network | 0.8431 | 0.6170 | 0.9149 | 0.0000 | 0.9149 |
|  | SVM | 0.9216 | 0.6011 | 1.0000 | 0.0000 | 0.9592 |
|  | Logistic Regression | 0.7059 | 0.6011 | 0.7234 | 0.5000 | 0.8193 |
| **Chemoradiotherapy Group** | Random Forest | 0.8148 | 0.9118 | 0.9000 | 0.6579 | 0.8630 |
|  | XGBoost | 0.8148 | 0.8940 | 0.9000 | 0.6579 | 0.8630 |
|  | Neural Network | 0.8148 | 0.8789 | 0.8857 | 0.6842 | 0.8611 |
|  | SVM | 0.8056 | 0.8594 | 0.8857 | 0.6579 | 0.8552 |
|  | Logistic Regression | 0.7778 | 0.8380 | 0.8286 | 0.6842 | 0.8286 |
| **All patients group** | Random Forest | 0.8239 | 0.9287 | 0.9145 | 0.5714 | 0.8843 |
|  | XGBoost | 0.8553 | 0.9267 | 0.9231 | 0.6667 | 0.9038 |
|  | Neural Network | 0.8428 | 0.9170 | 0.9573 | 0.5238 | 0.8996 |
|  | SVM | 0.8679 | 0.8909 | 0.9145 | 0.7381 | 0.9106 |
|  | Logistic Regression | 0.8428 | 0.8251 | 0.9231 | 0.6190 | 0.8963 |

Results are shown for the test sets of different groups (n=51 in radiotherapy alone group，n=108 in chemoradiotherapy group, n=159 in all patients group), for five classifiers: Logistic Regression, Random Forest, Extreme Gradient Boosting (XGBoost), Support Vector Machine (SVM), and a Neural Network. Sensitivity and Specificity refer to the true positive rate and true negative rate, respectively, for identifying patients with the toxicity.
